# SPISE and other fasting indexes of insulin resistance: risks of coronary heart disease or type 2 diabetes. Comparative cross-sectional and longitudinal aspects

**DOI:** 10.1080/03009734.2019.1680583

**Published:** 2019-11-07

**Authors:** Jan Cederholm, Björn Zethelius

**Affiliations:** a Department of Public Health and Caring Sciences/Family Medicine and Preventive Medicine, Uppsala University, Uppsala, Sweden;; b Department of Public Health and Caring Sciences/Geriatrics, Uppsala University, Uppsala, Sweden

**Keywords:** Coronary heart disease, HOMA, insulin clamp, insulin resistance, QUICKI, SPISE, type 2 diabetes

## Abstract

**Background:** Fasting insulin resistance indexes are used extensively nowadays. We intended to analyze a new recently presented fasting index, SPISE (sensitivity formula: 600 × HDL-cholesterol^0.185^/triglycerides^0.2^/BMI^1.338^), in comparison with three previously known fasting indexes, regarding correlation with the insulin clamp index, and for the predictive effects of future long-term risks of coronary heart disease (CHD) or manifest type 2 diabetes.

**Methods:** A total of 1049 71-year-old male subjects from the Swedish ULSAM study, median follow-up 8 years, were included. All subjects performed the euglycemic insulin clamp, and analyses of four fasting insulin resistance indexes: SPISE-IR (= 10/SPISE), QUICKI-IR, Log HOMA-IR, and Revised QUICKI-IR.

**Results:** Spearman correlation coefficients with the insulin clamp were 0.60–0.62 for all indexes. Area under curve at ROC analysis was 0.80 for SPISE-IR, and 0.84 for QUICKI-IR, Log HOMA-IR, and Rev QUICKI-IR. Adjusted hazard ratios per 1 SD index increase for long-term risk CHD were similar in all patients: 1.20–1.24 (*p* = 0.02–0.03). However, comparing the highest quartile (recommended to define insulin resistance) with the lower quartiles, SPISE-IR was the strongest and the only statistically significant insulin resistance index: HR 1.53 (*p* = 0.02). Adjusted odds ratios per 1 SD index increase for long-term risk of type 2 diabetes were fairly similar (*p* < 0.001) in all patients: 1.62 for SPISE-IR, 1.97 for QUICKI-IR and Log HOMA-IR, and 2.04 for Rev QUICKI-IR, and also when comparing the highest versus the lower quartiles: 2.8–3.1 (*p* < 0.001).

**Conclusion:** SPISE, easily applicable, performed equally well as other fasting insulin indexes previously recommended for clinical use, regarding correlation with the insulin clamp, and as predictor for future long-term risks of CHD or type 2 diabetes.

## Introduction

Several studies have indicated that increased insulin resistance is a predictor for the development of type 2 diabetes (1–4). It also significantly contributes to accelerated atherosclerosis as a risk factor for coronary heart disease (CHD) (5–7). The euglycemic insulin clamp technique is regarded as the reference method for an accurate assessment of *in vivo* insulin resistance (8–10). However, this method is laborious, expensive, and considered unsuitable for larger-scale or epidemiological studies. Other measures at the fasting state have been presented to be more clinically suitable and useful surrogate indexes of insulin resistance/sensitivity, like the homeostasis model assessment (HOMA-IR) index (11,12), Log HOMA-IR index (13–15), quantitative insulin sensitivity check index (QUICKI) (16), and Revised QUICKI index (17). However, indexes of insulin sensitivity are also available using 0-h and 2-h glucose and insulin values during the standard 75-g oral glucose tolerance test (OGTT), like the Cederholm index (18–20) and the Matsuda index (21,22). Furthermore, a new fasting insulin sensitivity index, SPISE (single-point insulin sensitivity index), has recently been introduced by the RISC and Beta-JUDO Investigators as an easily applicable tool in clinical practice, based on the ratio of triglycerides to high-density lipoprotein-cholesterol (TG/HDL) and body mass index (BMI) (23). After several repeated regression modelings, the best formula for SPISE was presented as: 600 × HDL^0.185^/TG^0.2^/BMI^1.338^.

The aim of this study was to evaluate the correlation between the resistance index SPISE-IR (defined as 10/SPISE) and the euglycemic insulin clamp, and to estimate the effect of SPISE-IR as a predictor for risks of future CHD and manifest type 2 diabetes. We also made a comparison with the indexes QUICKI, Log HOMA-IR, and Revised QUICKI regarding their correlations with the insulin clamp and as predictors of these outcomes.

## Material and methods

### Subjects

All men born between 1920 and 1924 in Uppsala, Sweden, were invited to a health survey in 1970 in which 2322 men (82%) participated – the ULSAM study (24). After 20 years, at 71 years of age, 1221 (73%) of the 1681 still-living subjects were invited for reinvestigation in 1991–1995, which constituted the baseline of this study (25). The study was approved by the Ethics Committee of the Faculty of Medicine at Uppsala University, and it complies with the principles of the Declaration of Helsinki. Written informed consent was obtained from all subjects. Patients with data available for all analyzed variables in this study numbered 1049 subjects. This sample was used to analyze the correlation between the euglycemic insulin clamp test and the fasting insulin indexes presented here, and to analyze these indexes as predictors of risk for ischaemic heart disease.

A subgroup was also created consisting of 1024 participants, after exclusion of those with manifest diabetes at baseline, and with data available for all analyzed variables. This subgroup was used to analyze the fasting insulin indexes as predictors of risk for development of type 2 diabetes. Type 2 diabetes at baseline was defined according to the 1999 World Health Organization criteria (26). Somewhat fewer inclusion criteria regarding the variables applied, as covariates in the regression analyses were used in this subgroup.

### Baseline investigations

Baseline investigations in 1991–1995 consisted of an euglycemic insulin clamp test, 75-g OGTT, HDL-cholesterol, triglycerides, body weight and height, non-esterified fatty acids (NEFA), cystatin C, microalbuminuria, systolic blood pressure under standardized conditions, and smoking present or not (25,27,28). BMI was calculated as weight/height squared (kg/m^2^). Microalbuminuria was measured as the urinary albumin excretion rate (mg/min). The Charlson comorbidity index was included for classification of a range (score) of comorbid diseases and conditions that may affect outcomes in prospective studies (29).

Glucose tolerance was assessed by 75-g OGTT, separated in time by 1 week from the euglycemic insulin clamp procedure (28). Blood samples for fasting concentrations were collected after overnight fasting, and blood samples were also collected at 2-h during the OGTT. Concentrations of plasma glucose were analyzed by the glucose dehydrogenase method (Gluc-DH; Merck, Darmstadt, Germany). Plasma immuno-reactive insulin (IRI) was determined with the enzymatic immunologic assay Enzymmun (Boehringer Mannheim, Mannheim, Germany) (27). These OGTT data were used to detect manifest diabetes at baseline of this study, and also for estimation of OGTT-based insulin resistance indexes in a previous study from our group (20).

### Insulin resistance measures

The euglycemic insulin clamp is considered gold standard for measurement of insulin sensitivity (1,8,27,28). Insulin was infused at a constant rate of 56 mU/min/m^2^, calculated to achieve nearly complete suppression of the hepatic glucose output (27,28). The target level of plasma glucose (measured every 5th minute during the 2-h clamp) was 5.1 mmol/L. Median was 5.1 mmol/L, 5th percentile 5.0 mmol/L, 95th percentile 5.4 mmol/L, and mean ± SD 5.2 ± 1.3 mmol/L. The insulin sensitivity index at the clamp (M/I) was calculated as glucose disposal rate (mg glucose infused/min/kg body weight) divided by the mean plasma insulin concentration × 100 (mU/L) during the last 60 min of the 2-h euglycemic insulin clamp. The inverse of the sensitivity index M/I is used here as the insulin resistance index, M/I-IR, defined as 10/(M/I).

SPISE (23) has been developed by the RISC and Beta-JUDO Investigators based on the ratio of triglycerides to HDL-cholesterol (TG/HDL) and BMI, with use of two included studies: the Relationship Between Insulin Sensitivity and Cardiovascular Disease study cohort (*n* = 1260; age, mean ± SD, 44 ± 8 years) and the Beta-Cell Function in Juvenile Diabetes and Obesity study cohort (*n* = 29; age, mean ± SD, 15 ± 2 years). All subjects underwent 75-g OGTT and euglycemic clamp tests for calculation of the clamp-derived sensitivity index M/I value. To refine the TG/HDL ratio, mathematical modelling was applied, including fasting TG, HDL, and also BMI, as compared to the clamp-derived M/I value, and each of several modelings was scored by the correlation coefficient with M/I and the specificity for insulin sensitivity identification. Based on several repeated modelings, the best formula for this new sensitivity index, SPISE, was then presented as: 600 × HDL^0.185^/TG^0.2^/BMI^1.338^. The inverse of this index, SPISE-IR, is used in this study as an insulin resistance index, and defined as 10/SPISE.

QUICKI has been introduced as a fasting index of insulin sensitivity, defined as 1/[log(fasting glucose) + log(fasting insulin)], where log is the natural logarithm, and glucose is measured in mmol/L and insulin in mU/L (16). The inverse of the sensitivity index QUICKI is used here as the insulin resistance index, QUICKI-IR, defined as 1/QUICKI.

Revised QUICKI has been presented as another fasting insulin sensitivity index based on glucose, insulin, and also additionally NEFA, and was defined as 1/[log(fasting glucose) + log(fasting insulin) + log(NEFA)] (17). The inverse of this sensitivity index was used here as the insulin resistance index, Rev QUICKI-IR, defined as: 1/Revised QUICKI.

HOMA-IR was described as a fasting index of insulin resistance by Matthews et al. with the formula: fasting glucose mmol/L × fasting insulin mU/L/22.5 (11). HOMA-IR has been shown to be highly correlated (*r* = 0.98) with a computer-derived HOMA-IR model (12) in the Framingham study (30). Only the former has been applied in this study as the basis for the insulin resistance index Log HOMA-IR, defined as the natural logarithmic transformation of HOMA-IR: log(fasting glucose × fasting insulin/22.5), as previously described by others (13,15).

### Follow-up and outcomes

All patients were followed-up after a median of 8 years, with up to a maximum of 10 years (censor date 31 December 2001). All events of fatal or non-fatal CHD, defined as ICD-10 codes I20–I25, were retrieved by data linkage with the Swedish Cause of Death and Hospital Discharge Registers, a reliable validated alternative to revised hospital discharge and death certificates (31,32). Type 2 diabetes during follow-up was defined as fasting plasma glucose ≥7.0 mmol/L in reinvestigations at age 77 years, or as new use of oral hypoglycaemic agents detected by questionnaire or in medical records during the follow-up period.

### Statistical analysis

Table 1 shows baseline clinical characteristics, given as means (SD) or frequencies (%), and also median values with interquartile range for the insulin indexes.

**Table 1.** Baseline characteristics in 1049 male subjects aged 71 years.

Clinical characteristics	All subjects (*n* = 1049)
Glucose 0 min, mmol/L	5.7 ± 1.4
Glucose 120 min, mmol/L	8.3 ± 4.1
Manifest type 2 diabetes, %	10.2
Insulin 0 min, mU/L	12.8 ± 8.3
Insulin 120 min, mU/L	70.1 ± 52.5
Clamp M/I-IR, 1/(mg/kg/min/mL)	2.7 ± 1.9; 2.1 (1.5–3.2)
SPISE-IR, 1/(kg/m^2^)	1.4 ± 0.3; 1.3 (1.1–1.5)
QUICKI-IR, 1/(mmol × mU/L^2^)	4.1 ± 0.6; 4.1 (3.8–4.5)
Log HOMA-IR, (L^2^/mmol × mU)	1.0 ± 0.64; 1.0 (0.64–1.4)
Rev QUICKI-IR, 1/(mmol^2^×mU/L^3^)^a^	4.0 ± 0.7; 4.0 (3.6–4.5)
Systolic BP, mmHg	147.1 ± 19
Hypertension treatment, %	33.4
BMI, kg/m^2^	26.3 ± 3.4
Total cholesterol, mmol/L	5.8 ± 1.0
LDL cholesterol, mmol/L	3.9 ± 0.9
HDL cholesterol, mmol/L	1.29 ± 0.35
Triglycerides, mmol/L	1.41 ± 0.69
NEFA, mmol/L^a^	0.52 ± 0.22
Microalbuminuria, μg/min	25.3 ± 95
Cystatin C, mg/L	1.24 ± 0.27
Smoker, %	20.7
Charlson comorbidity index, %	
Level 0	61.0
Level 1	20.8
Level 2	10.8
Level 3	4.5
Levels 4–7	2.9
A history of CHD, %	8.5

Data given as means ± SD or frequencies (%), and for the indexes also as median (interquartile range).

^a^Rev QUICKI-IR and NEFA were *n* = 1038 due to missing data for NEFA.

BP: Blood pressure; CHD: Coronary heart disease; HDL: High-density lipoprotein; IR: Insulin resistance; LDL: Low-density lipoprotein; M/I: Glucose disposal rate divided by mean plasma insulin during the clamp; NEFA: Non-esterified fatty acids.

#### Cross-sectional analyses at baseline

**Figure 1.**
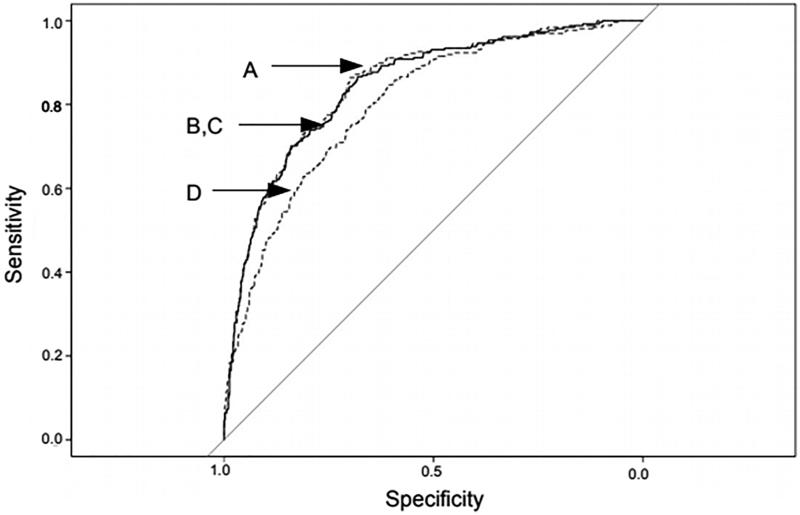
ROC curves for the highest quartile Q4 of M/I-IR (≥3.2) versus four fasting insulin resistance indexes, in 1049 71-year-old male subjects. Left dashed curve A: Rev QUICKI-IR; left solid curves B and C: QUICKI-IR and Log HOMA-IR; Right dotted curve D: SPISE-IR. See Table 2 for AUC values.

As Table 2 shows, Spearman correlation coefficients were used for comparison of M/I-IR with each of the four fasting insulin resistance indexes. Ninety-five percent confidence intervals of the coefficients were estimated by bootstrapping with use of the R package RVAideMemoire. Receiver operating curves (ROC) were used for comparison of the highest quartile of M/I-IR (≥3.2) with each of the four fasting resistance indexes, as shown in Figure 1. We estimated ROC area under curve (AUC) values for each comparison (Table 2). We also calculated the difference between AUC for SPISE-IR and AUC for each of QUICKI-IR, Log HOMA-IR, and Rev QUICKI-IR, and the significance of these differences. All ROC data were estimated with the R statistical package pROC.

**Table 2.** Spearman correlation coefficients, and ROC AUC values, comparing M/I-IR and each of the fasting insulin resistance indexes in 1049 71-year-old male subjects.

	Spearman coefficient (95% CI)	AUC value	Difference between AUC values		
Insulin indexes	M/I-IR in all patients	Q4 of M/I-IR	SPISE-IR	*Z* value	*p* value
SPISE-IR	0.62 (0.58–0.65)	0.801	–	–	–
QUICKI-IR	0.60 (0.56–0.65)	0.844	−0.043	2.3	0.02
Log HOMA-IR	0.60 (0.56–0.64)	0.844	−0.043	2.3	0.02
Rev QUICKI-IR	0.61 (0.57–0.65)	0.847	−0.046	2.6	0.01

Difference between AUC values compares AUC for SPISE-IR with AUC for each of the indexes QUICKI-IR, Log HOMA-IR, and Rev QUICKI-IR.

AUC: Area under curve (ROC; compares the highest quartile Q4 of M/I-IR [≧3.2] with each of the other indexes); CI: Confidence interval (based on bootstrapping; significance level all *p* < 0.001); IR: Insulin resistance; M/I: Glucose uptake per insulin unit at the clamp.

#### Longitudinal analyses of CHD risk

Cox regression analysis was used to estimate hazard ratios (HR) with the four fasting resistance indexes as predictors of long-term fatal or non-fatal CHD as outcome (Table 3). HR with 95% confidence intervals (CI) were given per 1 SD increase of each predictor index, allowing for a direct comparison between the strengths of the four hazard ratios. The *Z* statistic with its *p* values also indicates the association between predictor and outcome – the higher *Z* value, the stronger association. Covariance adjustment was performed with several important conventional cardiovascular risk factors and clinical characteristics: systolic blood pressure, smoking, cystatin C, albuminuria, Charlson comorbidity index, and a history of CHD. The proportional hazards assumption at Cox regression was confirmed with scaled Schoenfeld residuals, estimated with the R statistical package cox.zph. The likelihood ratio (LR) test indicates global model fit – the higher value, the better fit. Harrell’s C (concordance) measures discrimination between those who will have and those who will not have events – the higher value, the better discrimination, estimated with the R statistical package dynpred.

**Table 3.** The ability of four fasting insulin resistance indexes to predict long-term coronary heart disease, cox regressions in 1049 male subjects aged 71 years, 135 events during median 8 years of follow-up.

	All patients (*n* = 1049)					Q4 versus Q1–Q3^a^		
Insulin indexes^b^	HR (95% CI)	*Z* value^c^	*p* value	LR test^d^	Harrell’s C^e^	HR (95% CI)	*Z* value^c^	*p* value
SPISE-IR	1.20 (1.02–1.40)	2.2	0.03	44	0.64	1.53 (1.06–2.21)	2.3	0.02
QUICKI-IR	1.22 (1.03–1.45)	2.2	0.02	44	0.63	1.02 (0.70–1.51)	0.10	0.9
Log HOMA-IR	1.22 (1.03–1.45)	2.2	0.02	44	0.63	1.02 (0.70–1.51)	0.10	0.9
Rev QUICKI-IR	1.24 (1.04–1.49)	2.4	0.02	44	0.63	1.53 (0.99–2.37)	1.9	0.054

^a^HR for the highest quartile Q4 (*n* = 264) compared to lower quartiles Q1–Q3 as reference. Cut-off values for Q4 (75th percentile) of the indexes expressed per 1 SD were for SPISE-IR: 4.6; QUICKI-IR: 7.1; Log HOMA-IR: 2.2; and Rev QUICKI-IR: 5.1.

^b^Indexes expressed per 1 SD to allow for direct comparison between HR, adjusted for systolic blood pressure, smoking, cystatin C, albuminuria, Charlson comorbidity index, and a history of CHD.

^c^*Z* value: a higher value indicates stronger association between predictor and outcome.

^d^LR test measures global fit; the higher value, the better fit.

^e^Harrell’s C (concordance) measures discrimination between those who will have and will not have events.

CI: Confidence interval; HR: Hazard ratio.

#### Longitudinal analyses of risk for type 2 diabetes

Multivariable logistic regression was used to analyze the four fasting resistance indexes as predictors of long-term development of manifest type 2 diabetes as dependent outcome (Table 4). Odds ratios (OR) with 95% CI were given per 1 SD increase of each predictor index, making possible a direct comparison between the strengths of the four odds ratios, also indicated by *Z* statistics with *p* values. Adjustments were made for systolic blood pressure, smoking, and Charlson comorbidity index. The AIC value measures global model fit – the lower, the better fit. The C statistic measures discrimination between those who will have and those who will not have events – the higher value, the better discrimination.

**Table 4.** The ability of four fasting insulin resistance indexes to predict the long-term development of manifest type 2 diabetes, multivariable logistic regressions in 1024 male subjects aged 71 years, no diabetes at baseline 1991–95, 56 events of manifest diabetes during follow-up until 2001.

	All patients (*n* = 1024)					Q4 versus Q1–Q3^a^			
Insulin indexes^b^	Odds ratio (95% CI)	*Z* value^c^	*p* value	AIC value^d^	*C* statistic^e^	Odds ratio (95% CI)	*Z* value^c^	*p* value	*C* statistic^e^
SPISE-IR	1.62 (1.27–2.05)	3.9	<0.001	416	0.69	2.8 (1.6–4.8)	3.6	<0.001	0.68
QUICKI-IR	1.97 (1.47–2.64)	4.6	<0.001	409	0.72	3.1 (1.8–5.3)	4.0	<0.001	0.70
Log HOMA-IR	1.97 (1.47–2.64)	4.6	<0.001	409	0.72	3.1 (1.8–5.4)	4.1	<0.001	0.70
Rev QUICKI-IR	2.04 (1.48–2.81)	4.4	<0.001	402	0.72	2.9 (1.7–5.1)	3.8	<0.001	0.70

^a^Odds ratios for the highest quartile Q4 compared to the lower quartiles Q1–Q3 as reference. Cut-off values per SD for Q4 (75th percentile) were for SPISE-IR: 4.7; QUICKI-IR: 7.5; Log HOMA-IR: 2.2; Rev QUICKI-IR: 6.5. AIC values comparing quartiles were 418, 415, 415, and 409, respectively.

^b^Indexes expressed per SD to allow for direct comparison between OR, adjusted for systolic blood pressure, smoking, and Charlson comorbidity index.

^c^*Z* value: the higher the stronger association.

^d^AIC measures global fit; the lower value, the better fit.

^e^*C* statistic measures discrimination.

CI: Confidence interval.

All statistical analyses were performed with R version 3.5.3, 11 March 2019 (33). A two-sided *p* values <0.05 was considered statistically significant.

## Results

### Baseline characteristics

Mean ± SD values were 2.7 ± 1.9 units for insulin clamp M/I-IR, 1.4 ± 0.3 units for SPISE-IR, 4.1 ± 0.6 units for QUICKI-IR, 1.0 ± 0.64 units for Log HOMA-IR, and 4.0 ± 0.7 units for Rev QUICKI-IR (Table 1).

### Cross-sectional associations at baseline between clamp M/I-IR and insulin resistance indexes

Spearman correlation coefficients between insulin clamp M/I-IR and each fasting insulin resistance index were 0.62 (95% CI 0.58–0.65) for SPISE-IR, 0.60 (0.56–0.65) for QUICKI-IR, 0.60 (0.56–0.64) for Log HOMA-IR, and 0.61 (0.57–0.65) for Rev QUICKI-IR, all *p* < 0.001 (Table 2).

ROC curves for the highest quartile of M/I-IR (≥3.2) versus each of the four fasting resistance indexes are given in Figure 1. AUC values of the ROC curves were quite similar: 0.80 for SPISE-IR, 0.84 for QUICKI-IR and Log HOMA-IR, and 0.85 for Rev QUICKI-IR, (Table 2). Differences between AUC for SPISE-IR and AUC for each of the other indexes were slightly significant (*p* < 0.02–0.01).

### Longitudinal prediction of risk for CHD

The four insulin resistance indexes as predictors for long-term risk of fatal or non-fatal CHD estimated at Cox regression are shown in Table 3. The number of CHD events were 135 during median 8 years of follow-up, with 7625 person years at risk (Table 3). With each index introduced per 1 SD increase, and adjustments for systolic blood pressure, smoking, cystatin C, albuminuria, Charlson comorbidity index, and a history of CHD, SPISE-IR estimated in all patients disclosed a significant HR of 1.20 (1.02–1.40; *p* = 0.03), with a similar magnitude of HR as the other three fasting indexes, and with similar values of LR test and Harrell’s C. However, SPISE-IR was the only significant index (*p* = 0.02), and with the highest HR of 1.53, among all four fasting indexes, when analyzing the highest quartile versus the lower three quartiles.

QUICKI-IR and Log HOMA-IR were independent predictors of CHD in all patients, with the same HR 1.22 (1.03–1.45; *p* = 0.02), and the same LR tests and Harrell’s C. However, HR for these two indexes were non-significant when comparing the highest quartile versus the lower quartiles. Finally, Rev QUICKI-IR had a significant HR 1.24 (1.04–1.49; *p* = 0.02) in all patients, while HR for quartile 4 versus quartiles 1–3 disclosed an effect of only borderline significance.

### Longitudinal prediction of risk for manifest type 2 diabetes

The four fasting resistance indexes as predictors for risk of incident type 2 diabetes during follow-up were analyzed with multivariable logistic regression, with each index introduced per 1 SD increase to allow for direct comparison between strengths of the odds ratios (Table 4). In total, 56 new cases of type 2 diabetes were found during follow-up from baseline until 2001, in 1024 participants with no previous diabetes. Adjustments were made for systolic blood pressure, smoking, and Charlson comorbidity index.

SPISE-IR was strongly significant as predictor in all participants, with OR 1.62 (1.27– 2.05; *p* < 0.001), although slightly lower than OR of the other three indexes. Global fit was slightly less with a higher AIC value; the *C* statistic of discrimination was 0.69. Comparing the highest quartile versus the three lower, OR for SPISE-IR was strongly significant (*p* < 0.001), similar as for the other three indexes, with a *C* statistic of 0.68.

QUICKI-IR, Log HOMA-IR, and Rev QUICKI-IR showed similar OR in all patients, 1.97–2.04, and when comparing the highest quartile with the lower, 2.9–3.1, with similar AIC values. *C* statistics were 0.68–0.70.

## Discussion

The present large cohort study, including more than 1000 participants with data on fasting blood variables and gold standard euglycemic insulin clamp tests, showed cross-sectionally that the newly introduced fasting insulin resistance index SPISE-IR had a similar degree of Spearman correlation with the insulin clamp index M/I-IR as other previously used fasting resistance indexes, QUICKI-IR, Log HOMA-IR, and Rev QUICKI-IR. A meta-analysis in 2014 based on 120 articles has presented correlations between the insulin index at hyperinsulinemic euglycemic clamp tests and several fasting or OGTT-based insulin resistance indexes (15). Among the fasting surrogate indexes, they found the strongest correlation coefficients with Rev QUICKI-IR (*r* = 0.68), QUICKI-IR (*r* = 0.61), and Log HOMA-IR (*r* = 0.60), and these authors suggested to recommend these three as the most appropriate for use in large-scale clinical studies. However, they had provided no data on the recent SPISE-IR. Thus, our data imply that SPISE-R should be equally appropriate according to its Spearman correlation coefficient 0.62 observed here. The usefulness of SPISE-IR is underlined by the clinical practical advantage of no need to estimate less available data like fasting insulin or NEFA variables. An even better correlation between the insulin clamp test and SPISE-IR was reported in a recent Japanese study of 111 healthy non-diabetic men aged 30–50 years, with a Spearman coefficient of 0.69 (34).

The RISC and Beta-JUDO Investigators who introduced SPISE in 2016 have performed ROC analyses comparing the insulin clamp test M/I with either SPISE-IR, the fasting resistance index QUICKI-IR, or the fasting resistance index HOMA-IR (23), thus specifying insulin resistance among the total distribution of the insulin index (35,36). They found comparable ROC AUC values for all three indexes, 0.81–0.83, with no significant difference between them. ROC curves in our study comparing the insulin clamp index M/I-IR with the four fasting resistance indexes are presented in Figure 1. We found a mainly similar ROC AUC value for SPISE-IR in our study, 0.80, and also for QUICKI-IR, Log HOMA-IR, and Rev QUICKI-IR, 0.84 (Table 2). The study groups used by the RISC and Beta-JUDO investigators consisted of non-diabetic adults with mean BMI 26 kg/m^2^ and obese adolescents with mean BMI 38 kg/m^2^, while our study consisted of 70-year-old male subjects with mean BMI 26 kg/m^2^.

This study also showed that hazard ratios for long-term risk of future CHD in all patients were clearly significant and similar for SPISE-IR and the three other fasting indexes, QUICKI-IR, Log HOMA-IR, and Rev QUICKI-IR, and with similar Harrell’s C measuring discrimination (Table 3). However, comparing patients within the highest quartile of a resistance index with the patients within the lower quartiles, it was found that SPISE-IR had a clearly better capability to detect future risk of CHD. Values within the highest quartile are recommended (35) and often used (36) to characterize insulin resistance among the total distribution of a surrogate insulin index. Concerning OR for long-term risk of future type 2 diabetes, SPISE-IR generally had the same highly significant risk effect as the other three fasting indexes in all patients, and comparing the highest quartile with the lower, and with similar *C* statistic measuring discrimination (Table 4). Thus, our results are in agreement with the conclusion by the RISC investigators that SPISE-IR is performing equally well and as appropriately as the other previously recommended fasting insulin indexes at the meta-analysis in 2014. SPISE-IR is easily applicable in clinical practice with its use of an inexpensive routine fasting single-point blood sampling and BMI, and with a simply calculated index.

The RISC investigators also estimated ROC AUC values between the insulin clamp test and the OGTT-based insulin sensitivity index Matsuda-ISI, and found a somewhat higher AUC value of 0.86, with a significant difference between AUC values for Matsuda-ISI and SPISE (*p* = 0.006). Matsuda-ISI (= 1000/√ [0-min glucose × 0-min insulin × 120-min glucose × 120-min insulin]) and the corresponding Matsuda-IR (= 10/Matsuda-ISI) have been used as a well-performing surrogate insulin index in recent years (21,22). However, another OGTT-based insulin sensitivity index has been presented by our group, namely the Cederholm index (= [75,000/120 + (0-min glucose – 120 min glucose) × 0.19 × body weight kg × 180/120]/[(0-min glucose + 120-min glucose)/2]/log[(0-min insulin + 120-min insulin)/2]). This index (18,19) and the corresponding resistance index Cederholm-IR (= 100/Cederholm index) have been analyzed by our group and by others in previous studies (20,37,38). Matsuda-IR was reported to have a somewhat better simple correlation with the insulin clamp test than Cederholm-IR (21). Even if our previous survey using exactly the same sample of subjects as in the present study also found this (see the summary presented below), we found a better association between Cederholm-IR and the insulin clamp test according to Bland–Altman plot analysis.

A summary is presented below making a comparison between data from our present study and a previous study of our own (20). Using the same samples of 1049 and 1024 subjects in both, and with the same follow-up in both, it demonstrates Spearman correlation coefficients between M/I-IR and four insulin resistance indexes, HR per 1 SD insulin index increase for risk of CHD, and OR per 1 SD insulin index increase for risk of type 2 diabetes. This allows for a direct comparison of the predictive strength of different HR and OR, also with similar covariate adjustment in both studies (Table 5). It turns out that OGTT-based Cederholm-IR clearly is the strongest predictor of future CHD and diabetes (*p* < 0.001), and reasonably more able to measure combined hepatic and peripheral insulin resistance than fasting indexes like HOMA-IR. SPISE-IR had a lower correlation with M/I-IR, although similar predictive capacity as Matsuda-IR for risks of CHD and diabetes according to strengths of HR and OR. It cannot be excluded that SPISE-IR could mainly capture whole-body resistance, capturing combined hepatic and peripheral resistance, although measured at fasting.

**Table 5.** Comparisons of data from the present study and a previous study of our own (20).

Insulin indexes	Corr. M/I-IR versus index	Cox HR (95% CI)	*p* value	Logistic regression OR (95% CI)	*p* value	*C* statistic
Cederholm-IR^a^	0.71	1.31 (1.15–1.50)	<0.001	2.43 (1.87–3.15)	<0.001	0.83
Matsuda-IR^a^	0.76	1.23 (1.07–1.43)	0.005	1.68 (1.34–2.11)	<0.001	0.76
HOMA-IR^a^	0.60	1.18 (1.02–1.30)	0.03	1.27 (1.05–1.54)	0.01	0.71
SPISE-IR^b^	0.62	1.20 (1.02–1.40)	0.03	1.62 (1.27–2.05)	<0.001	0.69

^a^Previous study (20).

^b^Present study; HR and OR per 1 SD index increase, similar covariate adjustments.

CI: Confidence interval; Corr.: Spearman correlation coefficient; Cox HR: Hazard ratio at Cox regression; Log. Regr. OR: Odds ratio at multivariate logistic regression.

Strengths of this study are the large number of participants performing the euglycemic insulin clamp test, long-term follow-up of events, and outcomes retrieved by register linkages with reliable validated methods in Sweden (31,32). Using male subjects 71 years of age should mainly exclude the effects of hormonal variation. This age group is also sensible for estimation of risks for CHD and diabetes. A limitation may be that unmeasured covariates may have affected the results, although relevant covariates were extensively applied. BMI and blood lipids were not included as covariates in the present study, as these variables are included in the formula for SPISE.

The same results were generally obtained with both QUICKI-IR and Log HOMA-IR in this study regarding correlations, ROC AUC values, HR, and OR, in accordance with similar mathematical formulas: [log(fasting glucose) + log(fasting insulin)], and [log(fasting glucose × fasting insulin/22.5)]. We have included both these two surrogate indexes in this article in order to make the comparison between them obvious. However, our opinion is that in future studies it is not necessary to use both of them simultaneously, as they are identical and interchangeable.

The finding here, of several insulin resistance indexes to be strong predictors of CVD risk independently of several important conventional cardiovascular risk factors and comorbidities, underlines that insulin resistance also may be associated with other risk factors like endothelial dysfunction (39), chronic subclinical inflammation (40), impaired fibrinolysis, and hypercoagulability (41).

## Conclusion

In conclusion, the usefulness of surrogate indexes for insulin resistance in epidemiological studies depends on the strength of their correlation with criterion measures, but also importantly on the degree to which they predict future CHD and type 2 diabetes in long-term observational analyses. This large observational study including euglycemic insulin clamp tests has demonstrated that the newly introduced index SPISE-IR as well as previously recommended fasting indexes QUICKI-IR, Log HOMA-IR, and Rev QUICKI-IR all had a somewhat lower degree of correlation with the clamp resistance index than OGTT-based indexes like Cederholm-IR and Matsuda-IR. However, all had high ROC AUC values, with no pronounced difference between them. Although the OGTT-based Cederholm-IR had the strongest effect to predict future risks of type 2 diabetes and CHD among all analyzed indexes, SPISE-IR had a strongly significant effect to predict diabetes and also a significant effect to predict CHD, with a mainly similar predicting effect as the other three fasting insulin indexes. Finally, this was underlined regarding SPISE-IR when comparing the highest quartile of an index, indicating an insulin-resistant state, with the three lower quartiles. SPISE-IR turned out to have the strongest hazard ratio for risk CHD among the fasting indexes, and was the only one clearly significant (*p* = 0.02). The hazard ratio for risk of diabetes was also strongly significant (*p* < 0.001) with highest quartile of SPISE. Thus, this study indicates that SPISE-IR should be useful and even preferred among surrogate fasting insulin resistance indexes, with the advantage of being easily available in clinical practice, using only fasting blood lipids and BMI, and without the necessity to analyze insulin or NEFA variables. Likewise, SPISE-IR should be applicable in clinical studies on treatment of cardiovascular risk factors for risk prediction of CHD, type 2 diabetes (34), non-alcoholic fatty liver disease (42), and also possibly useful for complementary evaluation of insulin resistance in obese patients suitable for gastric bypass operations. Further clinical controlled studies should be valuable to validate the clinical utility of SPISE as a biomarker, to be used in prevention of future CHD and type 2 diabetes (34), as well as in the prevention of non-alcoholic fatty liver disease (42).
